# The acquisition of personal pronouns’ comprehension and production in French-speaking children: Toward the ability to embody characters’ perspectives in various pictured speech interactions

**DOI:** 10.1371/journal.pone.0324218

**Published:** 2025-09-22

**Authors:** Jean Xavier, Clément Villières, Camille Bélichard, Chloé Chêne, Nicolas Bodeau, Sébastien Fixary, Lucie Broc, Michel Fayol

**Affiliations:** 1 Department of Child and Adolescent Psychiatry, Henri Laborit Hospital Center, Poitiers, France; 2 CNRS UMR 7295, Cognition and Learning Research Center, University of Poitiers, Poitiers, France; 3 Faculty of Medicine and Pharmacy, University of Poitiers, Poitiers, France; 4 UNILIM - University of Limoges, Limoges, France; 5 Department of Child and Adolescent Psychiatry, Esquirol Hospital Center, Limoges, France; 6 CNRS LAPSCO lab, University of Clermont Auvergne, Clermont-Ferrand, France; Industrial University of Ho Chi Minh City, VIET NAM

## Abstract

Language acquisition involves the ability to switch perspectives according to the partners’ roles in the speech context. This ability, involving morphosyntactic and pragmatic aspects, is fundamental in the acquisition process of personal pronouns (PersP). Our aim is to analyze, in spoken French, the acquisition of comprehension and production of PersP according to different perspectives (1p, 2p, 3p) and functions [subject (S); direct object (DO) and indirect object (IO) clitics]. 110 children aged from 6 to 13 participated. Comprehension and production of PersP were evaluated through an experimental protocol consisting of various ecological situations of the children’s daily life, in a comic strip format. Sentences had two levels of difficulty: D1, involving S and DO; and D2 involving S, DO and IO PersP. We found an age effect on the scores for the two tasks. Scores for expected answers were higher in the comprehension (1) than in the production task (2). We identified a significant increase in the mean scores, after an estimated breakpoint of 8 years for (1), and after the two estimated breakpoints of 8 and 10 years for (2). In (2) we found significantly better performances for D1 than for D2 sentences. In D1, unexpected answers occur more significantly on the DO than on the S, and in D2 they occur first on the DO, then on the IO and finally on the S. In D1 sentences, the DO is pronominalized but challenging for gender; in D2 sentences it is mainly omitted, like for IO. After the age of 6, children’s performances in the acquisition of PersP’s comprehension and production increase with age. At the age of 8 for the comprehension task, and at the ages of 8 and 10 for the production task we identified a significant increase in the children’s performances. In this last task, the difficulties could be explained by its computational complexity in terms of morphosyntactic and pragmatic constraints The difficulties were focused on the use of object pronouns and mainly DO. We suggest that, in D2 sentences, children focus primarily on the partners of the interaction involved in the speech context (designated by S and IO), rather than on the object of the interaction (DO).

## Introduction

An important, prerequisite of language is that it must be acquired in an interpersonal situation, i.e., as a way of interacting with another person. It involves the identification of referents using linguistic expressions. This ability, allowing the pairing between referents and referential expressions, is fundamental in the acquisition process of personal pronouns (PersP).

PersP represent a class of grammatical units in which code and message overlap. Their general meaning cannot be defined without referring to the context in which they are embedded [1]. In a developmental perspective, PersP are firstly used in concrete situations of everyday life and then, out of real situations, in linguistic context. PersP use requires the speaker’s ability to integrate grammatical, semantic and discourse-pragmatic aspects to select the optimal referential expression. The fact that the development of this ability is poorly known and studied in French motivated this study. The aim of this study is to explore how French speaking children from 6 to 13 years old understand and produce PersP using comic strip format showing interactive situations between characters (e.g.,: a hairdresser and his customer).

### Morphosyntaxic aspects in PersP acquisition

French has a system of clitic pronouns with morphosyntactic properties such as dedicated positions in the functional structure of the sentence, movement processes and position constraints. PersP are defined considering their form (perspective, function, gender, number) and their order (position) in the sentence structure. First and second personal pronouns (1p and 2p) refer respectively to the speaker and to the addressee while the 3rd person (3p) is defined as referring to neither speaker nor addressee but to the non-participant excluded of the speech event [2,3]. In French language, according to Benveniste (1966) [4], the reference of 3p object clitic to identify the non-participant, must be established via syntax/discourse. In contrast, 1p and 2p forms, as deictics are context-bound respectively referring to the speaker and to the addressee, according to their role in the speech act. Syntactically, this role is in correspondence with the function of its referent in the sentence, as subject (S), direct object (DO) and indirect object (IO) [Table 1 and examples below]. S and DO PersP are considered clitics due to their phonological, morphological and syntactic properties as weak elements [5].

**Table 1:** An overview of the French pronominal systems.

Person/ Perspective		Subject	Direct Object pronoun	Indirect object pronoun
Singular	**1**^**st**^ **person**	Je	Me	Me
	**2**_**nd**_ **person**	Tu	Te	Te
	**3**_**rd**_ **person (m)**	Il	Le	Lui
	**3**_**rd**_ **person (f)**	Elle	La	Lui
Plural	**1**_**st**_ **person**	Nous	Nous	Nous
	**2**_**nd**_ **person**	Vous	Vous	Vous
	**3**_**rd**_ **person (m)**	Ils	Les	Leur
	**3**_**rd**_ **person (f)**	Elles	Les	Leur

Concerning DO clitics, while 1p and 2p (*me, te, nous, vous, les*) are marked for number, 3p personal pronouns (*le, la, les*) are morphologically marked for gender as well. IO clitics (*me, te, lui nous, vous, leur*) are only marked for number [6,7].

In spoken-French, the usual word order is SVO (Subject-Verb-Object) in declarative sentences and interacts with subject and object clitic pronouns. Subject clitics occur in the canonical SVO order in that they immediately precede the verb (example 1a), while the object usually occurs after the verb.

Lexical objects come after the verb whereas, with object complement pronominalization and cliticization, word-order constraints are more complex: pronominal objects are clitics that directly precede the verb, as illustrated in sentences (1b) and (2a, 2b). In addition, in a sentence with two pronominal objects, the relative position of DO and IO depends upon the person of the IO (see ex. 2a, 2b).

With one pronoun, SOV is the only possible order as in:

Ex 1. Pierre lance le ballon (‘Peter throws the ball’) [S V O].

a. Il lance le ballon (‘He throws the ball’) [S V O]

b. Pierre le lance; *Lit: ‘Peter it throws.’* (’Peter throws it’) [S DO V].

With two pronouns, IO is before DO in the 1p and 2p (2a) and DO is before IO in the 3p (2b):

Ex. 2. Pierre lance le ballon à Paul

a. Pierre te le lance; *Lit: Peter you it throws* (‘Peter throws it to you’) [S IO DO V].

b. Pierre le lui lance *Lit: Peter it him throws* (‘Peter throws it to him’) [S DO IO V].

Numerous studies grounded in spontaneous and elicited production uncovered that French personal subject pronouns are acquired very early, by the age of 2 [8–12]. Studying the comprehension and the production of subject clitic pronouns in 2-year-old French acquiring children, Veneziano et al. (2022) [13] found that, for subject clitic pronouns, the results of comprehension and production were closely related.

Jisa et al. (2010) [14] show that 5- and 7-years old children construe narrative events with the primary character as topic and subject, according to the ‘thematic subject strategy’ [15]. Following this idea, a given referent expression corresponds to a referent/agent which is the one who acts and which is the topic of the proposition construed about him [16].

In contrast with the regular production of pronominal subject clitics, pronominal object clitics are commonly absent from the first utterances produced by French-speaking children [17–19]. Children produce object clitics more systematically around the age of 3 [20] and omissions remain visible later than for subject clitics [21]. This omission of object clitics is an optional phenomenon and omission rate varies across different age groups (For a review see [22]). According to this review, for 6 years old children, DO clitics production rate is between 70–93%, the rate of DO clitics omission is between 0–10% and the rate of production of full DP (non-pronominalization) varies between 7–12%.

Language development and PersP acquisition is affected by computational aspects of grammar in terms of syntactic complexity involved in each construction [23–27].

Zesiger et al. (2010) [19] examined the development in comprehension and production of the pronominal system, including S and DO clitics, in a group of French-speaking children between 4–6 years old. The authors found 1) poorer performances while using object clitics than subject clitics with more omissions of the object clitic than the subject clitic in young children (21% vs. 7.8%); 2) difficulties in the use of pronoun gender in a greater proportion in the younger children. Among the 3p, DO clitics (*le, la*) have shown to be particularly affected in production by younger children. As reviewed by Tuller et al. (2011) [7], several factors may explain this difference: i) 3p object clitics are not deictic elements: their reference has to be found in the discursive context, 2) they co-occur with a nominative clitic and 3) 3p object clitics are marked for both gender and number. Leger et al. (2015) [28] found better performance in comprehension than in production of object PersP in French-speaking children aged 4–9. Furthermore, the authors explored the processing of 3p forms of DO clitics during an elicited production task administrated amongst French-speaking children from 4 to 9 years old. They found a significantly lower production rate of object clitics in the younger children (4–6 years old) which could, by contrast, process them in comprehension. Other studies report high rates of omissions of DO clitics between 2 and 4 years old [9,19,20,29–32].

Bello & Pirvulescu (2022) [33] compared the production of DO and IO clitics based on induced and spontaneous data in French-speaking children from 3 to 5 years of age. The authors used the sentences including either DO or IO. They found (1) significant omissions of both clitics in children in comparison to adults and (2) an asymmetry between the acquisition in DO and IO clitics. In spontaneous speech, IO clitics appeared later than OD clitics. In induced production, IO clitics were omitted at a higher rate than OD clitics.

### The PersP’s acquisition, from a discourse/ pragmatic point of view

The appropriate use of referring expressions involves the ability to consider the mental states of the partners involved in the speech context. This common ground termed ‘mutual knowledge’ [34] corresponds to the beliefs and assumptions shared by the speaker and addressee [35,36]. The pairing between referent expression and the different dialogue roles emphasizes the underlying cognitive dimension involved in the grammar development [37]. It corresponds to the shifting reference [4,36,38–40] i.e., the ability to switch perspectives according to the different roles occupied by the partners in a speech context. Loveland (1984) [41] insists on the spatial aspects of pronoun acquisition and the importance of seeing different speakers interacting in the children’s environment in order to understand perspective shifting. Concerning the order of emergence of 1p, 2p, 3 p’s comprehension and production, various hypothesis have been put forward:

- The ‘speech role hypothesis’ [40] which states that children, from the onset, grasp the relationship between pronouns and speech roles.

- The ‘name hypothesis’ [40,42]: young children may ignore the shifting reference of pronouns and treat them like proper names, which leads to pronoun errors and reversals.

- The ‘person-role hypothesis’ [43]: children learn the pronouns referring to themselves before mastering the pronouns referring to others.

Girouard et al. (1997) [44] lead a longitudinal study on the knowledge of 1p, 2p, 3p amongst French-speaking young children as addressed listeners and speakers. The results indicated that the mastery of pronouns did not follow the developmental sequence predicted by the speech-role hypothesis; they provided evidence for the person-role hypothesis only when children were speakers, and partially supported the name hypothesis. They found that these three pronouns were understood at about the same time (between 21 and 22 months). They noticed that 1p appears in children’ speech around the age of 26 months. Then 2p and 3p were produced respectively around the age of 29 months. Finally, by testing children as non-addressed listeners, they found that they were delayed in the comprehension of 3p.

Concerning pronoun reversal, even if it rarely occurs in typical development, Chiat (1981) [45] proposed the ‘perspective shifting hypothesis’. According to Morgensten et al. (2013) [46], this hypothesis supports the idea of a transition period during which pronoun reversal would be a way for children to concretely experiment shifting mental perspective. Piaget (1967) [47] described the phenomenon of perceptual egocentrism as a pivotal factor in the development of a child’s cognitive skills. He reported that substantial progress occurs around the ages of 7–9. Then, young children would be egocentric communicators who, in both speaker and listener roles, see things from their own perspective. Regarding language acquisition, Piaget (1962) [48] used the term ‘centrism’, meaning that the child cannot differentiate his perspective and the perspective of others. By contrast, Bezuidenhout et al. (2013) [49] noticed that children (even ones under the age of 3) are not trapped in their own egocentric world. In addition, studies of spontaneous conversational discourse have shown that children use appropriately the full range of personal pronouns by the age of 3 (For a review see [50]). By contrast, Hendriks and Spenader (2006) [51] argue that children continue to perform poorly on the interpretation of pronouns, even up to the age of 6 and a half.

### Synthesis and hypothesis

A French-exposed child must learn how to identify a pronoun’s person, gender, case, and number to correctly use the pronoun system according to the speech context, i.e., to the speech situation. The fact that comprehension and production of PersP appear early in speech development does not entail that children can use them correctly in different situations. It requires the ability to shift perspectives according to his/her and others speech roles, particularly in a narrative context involving S, DO and IO PersP, with several referents (see example 3).

Example 3:

In a game situation with Peter Jenny and Paul,

- Peter throws the ball to Paul; Peter says to Jenny:

Je la lui lance (‘I throw it to him’). The referent of him (IO) and I (S) are respectively Paul and Peter. The referent of it (DO) is the ball.

- Jenny throws the frisbee to Peter; Jenny says to Paul:

Je le lui lance (‘I throw it to him). The referents of I (S) becomes Jenny and the referent of him (IO) Peter becomes you. The referent of It (DO) is the Frisbee knowing that 3p DO is marked for gender (see above).

In a developmental perspective, experiencing such situations, i.e., such speech contexts allow children to practice syntactic rules and perspective shifting involved in the use of PersP. In these situations, children are speakers, listeners, as well as witnesses of the interaction between different speakers.

The literature mentioned above, reports: (1) a better performance in comprehension than in production of object PersP in French-speaking children aged 4–9 (2) an acquisition asymmetry between subject and object PersP [21].

In addition, the data obtained from previously mentioned studies have been gathered with different methodologies: spontaneous production does not offer the opportunity to study the use of PersP with the possibility for children to make errors. Moreover, the use of somewhat too artificial settings do not include interactions between several partners.

Finally, few studies integrate comprehension and production tasks and to our knowledge, there is no recent studies focusing on the acquisition process of PersP in French-speaking children and especially for children from the age of 6 years (for recent studies see Veneziano et al. 2022, Salazar Orvig et al. 2021; Silva Genest et al. 2021) [13; 52,53].

PersP play a central role in the communicational dimension of language. Furthermore, they are weak elements in oral language, especially the object (le, la, les, lui leur), some of them not even appearing in oral language [5]. Their use thus benefits from the acquisition of written language which develops in the context of school education, in children from 6 years old.

Our study focuses on the acquisition of PersP in French-speaking from 6 years old, testing children’s comprehension and production of PersP in a controlled setting. It involves various speech situations, different perspectives (p1, p2, p3) and functions in sentences (S, DO, IO), in order to determine more precisely children’s level of PersP mastery. We developed a single elicitation protocol to analyze the developmental course of PersP’s comprehension and production for children from 6 to 13 years of age. Participants were presented with illustrations representing two characters interacting while a third (with different symbolism) is in the observer position (see Method). The participant had to place himself/ herself in the position of several characters in various speech situations.

### We formulated the following hypotheses

i) Performances are better in comprehension than in production of PersP.
ii) There will be a positive developmental age effect on PersP’s comprehension and production.
iii) Performances in production of S would be higher than performances in production of DO and IO PersP.
iv) The rate of expected answers is higher for p1 and p2 perspectives than for p3.

## Materials and Methods

### Participants

110 children, aged 6–13 years, enrolled in ordinary schools took part in this study in October and November 2022. Inclusion criteria were as followed: to be enrolled in their age group, to have no learning or academic difficulties requiring intervention by the Special Needs Department for pupils with difficulties, and to be French native speakers. The experiment was carried out in accordance with the ethical standards of the 1964 Declaration of Helsinki. Part of the participants was only involved in this experiment. Another part was involved in another protocol for which the experiment constituted the first task. All the parents were informed, by means of an information letter, about the setting up of the study and its objectives. They all completed a written consent form authorizing their child to take part in the study. The processing of the data collected was validated as compliant with 1) the General Data Protection Regulation under reference 202347 and 2) the ethics committee registration number: 21.05224.011364; National number: 2021-A00359-32 (for participants included in the other protocol).

Table 2 shows the characteristics of the participants.

**Table 2:** Characteristics of the participants.

Age range	N	mean	Standard deviation
6 - 7	17	6.54	0.27
7 - 8	21	7.45	0.35
8 - 9	14	8.51	0.22
9 - 10	26	9.46	0.24
10 - 11	26	10.42	0.3
11 - 12	2	11.33	0.47
12 - 13	4	12.56	0.1

### Tasks

Comprehension and production of PersP were evaluated through an experimental protocol consisting of various speech contexts in a comic strip format. The tasks were adapted from the “Adverbial Pronoun Bingo” test [54] (see supplementary files). We used 20 comic strips – 10 for comprehension, 10 for production- depicting various daily life situations involving characters of different ages and genders. According to the development of children’s syntactic abilities, tasks involved two levels of sentence difficulties depending on the presence of DO only [D1, level difficulty 1, e.g., ‘He pulls me’] or on the presence of DO and OI [D2, level difficulty 2, e.g., ‘He gives it to him’]. Within each task containing 10 comic strips, randomly shown, 5 were of difficulty 1 (D1) and 5 were of difficulty 2 (D2). For each comic strips, a short scenario provided a description for the scene observed. In addition, a third character observing the scene was added to each board.

In each task (comprehension and production), an example was given to the child before starting the tasks, to make sure that the participant had understood what was being asked of them. If necessary, the experimenter re-explained the instructions to the participant.

**Fig 1:**
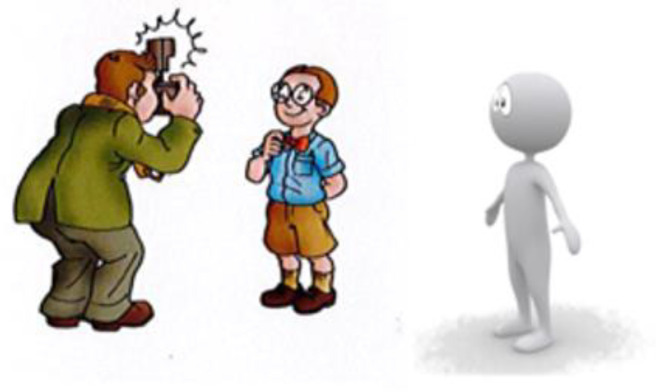
The picture of a photographer taking a picture of a little boy.

For the comprehension task, the instructions were as follows (instruction are in French in supplementary files): ‘I’m going to show you some pictures that tell short stories. For example, in this picture (Fig 1) there is a boy (the experimenter shows the boy), there is a photographer (the experimenter shows the photographer) and there is someone watching (the experimenter shows the person watching). Each person can speak. Show me who is saying: je le prends en photo ‘I’m taking a picture of him’ (expected answer: the photographer); ‘He is taking a picture of him’ (expected answer: the person who is looking); ‘Il me prend en photo ‘He’s taking picture of me’ (expected answer: the boy).

An example of picture in the comprehension task. The experimenter showed to the child the different characters: the little boy, the photographer and the character watching the scene.

**Fig 2:**
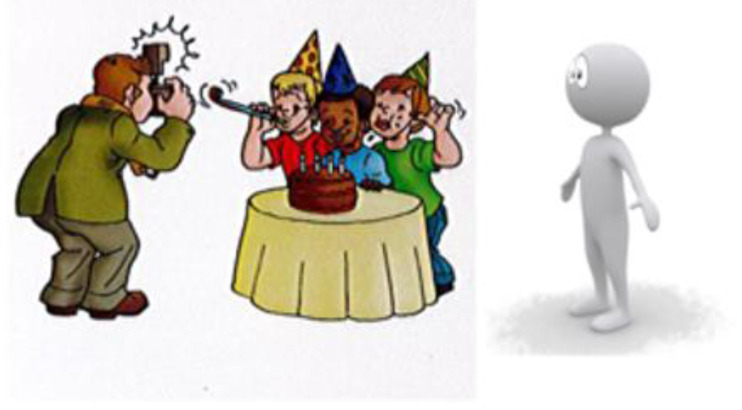
The picture of a photographer taking pictures of children. An example of picture in the production task. The experimenter showed to the child the different characters: the photographer, the children and the character watching the scene.

For the production task, the instructions were as follows: I’m going to show you some pictures that tell short stories. For example, in this picture (Fig 2), there is a photographer (the experimenter points to the photographer with his forefinger), there are children (the experimenter points to the children with his forefinger) and there is someone looking (the experimenter points to the person looking with his forefinger). What do you think the photographer is saying? (Expected answer: Je les prends en photo; ‘I’m taking a picture of them’); what are the children saying? (Expected answer: il nous prend en photo; he is taking a picture of us’), what is the person who watching saying? (Expected answer: il les prend en photo, He is taking a picture of them’).

### Experimental procedure

The experimenter met the participants in the school setting, on a one-on-one basis. The experimental sessions lasted between 20–45 minutes. According to the developmental perspective mentioned above, the task of understanding pronouns was always proposed before the task of producing pronouns. We had to make sure that the pupils understood the situations before questioning their productions. Between the two tasks, we included a distractibility task (backwards digit span task, [55]). Each child was confronted with all three-speech roles presented randomly for each context.

The instructions for the distractibility task are as followed: ‘Now I’m going to tell you some numbers and you’re going to have to repeat them backward. For example, if I tell you 6 - 9, you have to repeat 9 - 6. The list of numbers increases as you go along. We start with two numbers as in the example and then move on to 3, 4, 5, etc. Have you understood correctly?’

The experimenter recorded all the answers given by the participant in each task (comprehension, production, and distractibility task) during the experiment, in an Excel spreadsheet.

### Coding: Comprehension and production task

Each answer produced by the children in the comprehension and production tasks were coded as expected answer or unexpected answer. Whether or not the answer is considered as expected depends on the ability of the participants to embody the different characters’ discourse, adopting each of the three perspectives.

Expected answer: pronominalization of the speaking referent with respect of its functions (S, DO, IO), number, gender, position, and perspective (1p, 2p, 3p) in the speech context. Table 3 shows the distribution of the number of each perspective according to each function.

**Table 3:** Number of each perspective according to PersP functions.

	S**	DO**	IO***
1p	10	5	5
2p	0	0	4
3p	20	25	6

* S: Subject, * * Direct object, * * * Indirect Object.

Unexpected answer: non-pronominalization of the referent (noun phrase), omission or errors in gender of PersP in the speech context. Table 4 shows types of unexpected answers in production. It concerned the subject personal pronoun, either the DO in D1 sentences and the DO or IO in D2 sentences.

**Table 4:** Production task: examples of unexpected answers.

Sentence Difficulty	The error relates to	Category of error	Unexpected answer	Expected Answer	Unexpected answers(M, SD)
**D1** (S + DO)*	Subject	Gender	Il le borde ‘He tucks him in’	Elle le borde ‘She tucks him in’	0.13, 0.33
		Noun phrase***	Le coiffeur les coiffe ‘The hairdresser styles her hair’	Il les coiffe ‘He styles her hair’	0.08, 0.45
	DO	Gender	Il le gronde ‘He is telling him off’	Il la gronde ‘He is telling her off’	0.30, 0.66
		Noun phrase***	Il pousse le garçon ‘He pulls the boy’	He pulls him ‘Il le pousse’	0.18, 0.65
		Omission	Il tire ‘He is pulling’	Il le tire ‘He is pulling him’	0.15, 0.49
**D2** (S + DO + IO**)	Subject	Gender	Elle les lui coiffe ‘She is styling it for her’	Il les lui coiffe ‘He is styling it for her’	0.16, 0.40
		Noun phrase***	La maman les lui lave ‘The mum washes them for her’	Elle les lui lave ‘She washes them for her’	0.12, 0.63
	DO	Gender	Il le lui remet ‘He is awarding it to him’	Il la lui remet ****	0,25, 0.61
		Noun phrase***	Je lui sers une pizza ‘I am serving him a pizza’	Je la lui sers ‘I’m serving it to him’	3.20, 3.17
		Omission	Il me coiffe les cheveux He is styling my hair	Il me les coiffe ‘He is styling it for me’	8.24, 3.19
	IO	Gender	None	–	0
		Noun phrase***	Il le dessine pour le garçon ‘He is drawing it for the boy’	Il le lui dessine ‘He draws it for him’	0.10, 0.41
		Omission	Il le dessine ‘He is drawing it’	Il le lui dessine ‘He is drawing it for him’	4.65, 1.76

* Direct object ** Indirect object *** non-Pronominalized **** In French the use of 3p DO is marked for gender.

**Fig 3:**
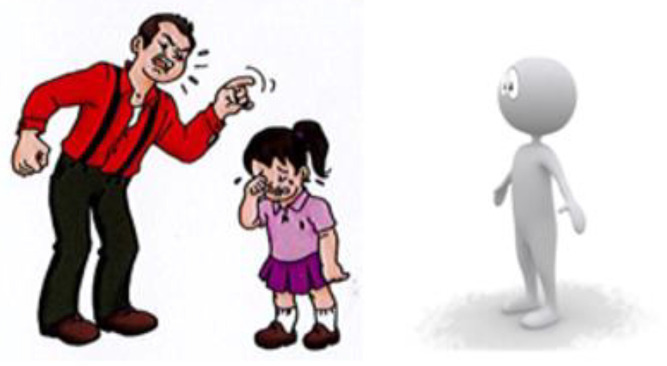
The picture of a father who is getting cross at his child.

In addition, for the production task, some answers were coded as expected answers with a change of perspective concerning the addressee. For example, the following picture (Fig 3) shows a father getting cross at his child. A 3rd person is watching the scene.

An example of picture in the production task. the experimenter showed to the child the different characters: the father, the children and the character watching the scene.

The participant is asked what the father is saying. The expected answer is ‘Je le gronde’ (‘I’m telling him off’). If the participant answers ‘Je te gronde’ (‘I am telling you off’), he has given an expected answer with a change of perspective on the DO.

The coding was carried out as followed

In the comprehension task, according to the level of difficulty of the sentences (D1 or D2), the answers given were coded 1 for an expected answer vs 0 for an unexpected answer. The highest number of expected answers in comprehension was 15 for D1 sentences and 15 for D2 sentences.

In the production task, according to the level of difficulty of the sentences (D1 or D2), the answers given were coded: 0 for unexpected answers vs 1 for expected answers.

In addition, concerning unexpected errors in the production task, we recorded the type of errors depending on whether they relate on (S, DO and IO), number, gender, perspective (1p, 2p, 3p) or non-pronominalization.

We recorded separately the expected answers with a change of perspective.

### Statistical analysis

We performed statistical analyses using R software version 4.2.2.

Individual scores – the sums of correct answers- were computed for each task, and the following variables were entered in the analysis: developmental age, expected answers, expected answers with perspective change, unexpected answers, characteristics of personal pronouns (perspectives, functions, gender, number).

In a first step, a descriptive analysis was carried out to describe the population: frequencies for qualitative variables, min, max, median, mean and standard deviations for quantitative variables. In a second step, a bivariate analysis was performed to test the association between pairs of variables.

For associations between quantitative variables, Spearman’s Rank Correlation Coefficient was used because we were looking for a monotonic relationship between variables.

For group comparisons, Student’s t-test was used with a correction (Welch’s correction) in the case of unequal variances. In order to compare scores for the same respondents, (for example, when comparing D1 scores and D2 scores) we used a paired version of the t-test.A significance level of 0.05 was used for judging the significance of difference, and all tests were bilateral.

## Results and discussion

Table 5 shows the scores obtained by participants for both tasks, according to the two levels of difficulties of sentences (D1 and D2).

**Table 5:** Performances of the children in the comprehension and the production task.

	Comprehension task	Production task		
Sentence: level of difficulty	Expected answers (M, SD)	Expected answer (%) (M, SD)	Expected answers with change of perspective (%) (M, SD)	Unexpected answers (%) (M, SD)
D1 (S DO)	**98.1%** 14.71, 1.21	**84,5%** 12.68, 3.34	**7,7%** 1.15, 2.53	**7.6%** 1.14, 2.04
D2 (S DO IO, S IO DO)	**97.3%** 14.59, 1.40	**7.5%** 1.13, 2.14	**0.8%** 0.12, 0.4	**91.9%** 13.8, 2.31

S: Subject; DO: Direct object; IO: Indirect Object; M = Mean, SD = Standard Deviation.

For D1 and D2 sentences, we found that the scores obtained by the participants are higher in the comprehension task compared to those of the production task.

### Comprehension task (Table 5)

First, we found that participants had high scores in the comprehension task and, using a paired t-test, we did not find significant differences between scores obtained in D1 in comparison with those obtained in D2 (t = 1.44, p = 0.1525).

Furthermore, the number of expected answers was analyzed comparing ages of the participants according to the level of sentences’ difficulties.

We found a significant positive correlation (Spearman’s rank correlation) between age and performances in the comprehension task (rho = 0.39, p < 0.001). We also computed, for different age thresholds, Wilcoxon’s effect sizes when comparing scores between age groups (e.g., scores <7 y/o vs >=7 y/o, < 8 y/o vs >=8 y/o, etc.). The maximum effect-size (0.42) was found for the 8 years cut-off point meaning that it is the age that separates best the scores among respondents.

### Production task (Cf. Table 5)

We found 84.5% of expected answers for D1 and 91.9% of unexpected answers for D2 sentences. Participants gave more expected answers (84.5%) than expected answers with a change of perspective (7.7%). Comparing scores between D1 and D2, using paired t-tests, we found:

- For expected answers, significantly higher scores for D1 (M = 12.68; SD = 3.34) than for D2 sentences (M = 1.12; SD = 2.14) [t = 33.9, p < 0.001].

- For expected answers with change of perspective, significantly higher scores for D1 than for D2 sentences (t = 4.3; p < 0.001)

- For unexpected answers, significantly higher scores for D2 than for D1 sentences (t = − 48.1; p = 0).

Furthermore, we found a significant positive age effect for expected answers (rho = 0.35; p < 0.001) and a negative significant age effect for unexpected answers (rho = − 0.28; p = 0.003). We also computed, for different age thresholds, Wilcoxon’s effect sizes when comparing scores between age groups. The maximum effect-sizes respectively (0.32 and 0.31) were found for the 8 years and the 10 years cut-off points.

### Analysis of unexpected answers concerning PersP’s function (S, DO IO) and according to the difficulty levels of the sentences

Table 4 shows the unexpected answers given by the participants.

Since the S, DO and IO scores have been calculated for each patient, these scores have been compared using paired t-tests.

For D1,

only S and DO scores are available and we found that the number of unexpected answers occurring on DO (M = 1.37, SD = 1.83) is significantly higher than those occurring on S (M = 0.67, SD 1.62) [t = 4.8, p < 0.001].

Concerning the unexpected answers on DO, they are related first on the use of gender than on the non-pronominalization or on the omission of DO.

For D2,

we had 3 groups to compare: DO vs S, S vs IO and IO vs DO. So we used an adjustment of the p values to account for these multiple tests.

We found that:

- The number of unexpected occurring on DO (M = 11.7, SD = 2.73) is significantly higher than of unexpected answers occurring on S (M = 0.74, SD = 1.69) [p < 0.001].

- The number of unexpected answers occurring on IO (M = 6.33, SD = 1.78) is significantly higher than the number of errors occurring on S (M = 0.74, SD = 1.70) [p < 0.001]

- The number of unexpected answers occurring on DO (M = 11.7, SD = 2.73) is significantly higher than the number of errors occurring on IO (M = 6.33, SD = 1.78) [p < 0.001].

Concerning the unexpected answers on DO, they are related first on the omission of DO and then on the non-pronominalization of DO.

- For IO, unexpected answers are mainly related on its omission (M = 4.65; SD = 1.76).

### Analysis of the scores of expected answers according to PersP perspective and function of PersP, considering the difficulty levels of the sentences

For D1 sentences:

In S, scores of expected answers for 3p perspective (M = 87, SD = 20.8) are significantly higher than those with 1p perspective (M = 79.6, SD = 30.5) [t = −3.6, p < 0.001].

In DO, scores for 3p perspective (M = 81.4, SD = 24.5) are significantly lower than those for 1p perspective (M = 90.9, SD = 21.3) [t = 6.4, p = 0].

For D2 sentences,

In S, scores for 3p perspective (M = 8.5, SD = 17) are significantly higher than those for 1p perspective (M = 5.6, SD = 13.8) [t = 61.9, p = 0.056].

In DO, we do not have results concerning perspective because there was a majority of 3p.

For IO, scores were difficult to analyse because expected answers were very few as well as 2p perspectives.

**Fig 4:**
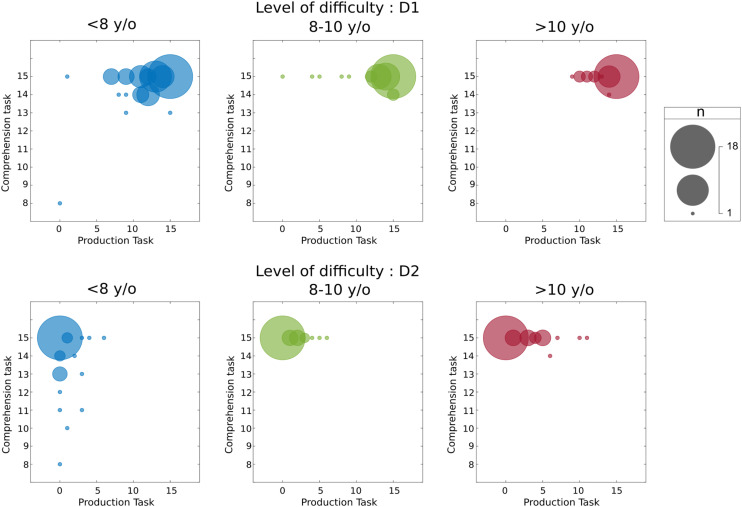
The relationship between comprehension and production task according to age and the two levels of sentences.

Fig 4 illustrates the relationship between comprehension task and production task according to ages and to the two levels of sentences D1 and D2.

We plot weighted scatterplots where each dot corresponds to a specific score combination (0–15) and its size reflects the number of individuals. Separate panels are provided for the age groups <8, 8–10, and >10 years.

In D1, the two tasks are both well succeeded with results which appear related. There is a dispersion of the results, above all in production, decreasing with age, from 8 and 10 years old. From the age of 8 comprehension task was succeeded and performances in the production task increased with age.

Conversely, in D2 results obtained in each of the two tasks are dissociated. For the group of children < 8, the production task appears to be very difficult (the results obtained are from 0 to 6). In comparison, the comprehension task seemed better succeeded with an important spread of the results (from 8 to 15). From the age of 8, the comprehension task was succeeded, contrary to the production task which remains difficult but with results that tend to improve.

The goal of our study was to explore the acquisition process of PersP comprehension and production in French-speaking children, from age 6–13. We used an experimental protocol, exploring the issue of reported speech, to test the syntactic and pragmatic cues occurring in PersP acquisition. This protocol consists of various ecological situations of the children’s daily life presented in comic strip form. Following a developmental perspective, we used (1) the comprehension task before the production task and (2) sentences with two levels of syntactic difficulty. The first one, D1, involves Subject (S) and direct object (DO); the second one, D2, involves S, DO and indirect object (IO).

Concerning the performances in the comprehension task we found an age effect. Exploring how scores of expected answers change with age, we identified a significant increase in the mean scores, after an estimated breakpoint at 8 years. This result illustrated is in Fig 4 where, in D1 sentences, we have a dispersion of results in comprehension decreasing at 8 years and at 10 years. In the comprehension task, participants had high scores not significantly different between D1 and D2 sentences. However, in Fig 4 the dispersion of the results appears to more important in D2 than in D1 and seems to decrease from the age of 8. The increase in performances in D2 from this age aligns with Frizelle et al. (2025) [56]. The authors reported an increase with age in the understanding of complex syntax in children between 5 and 9 years old.

In compliance with the results reported by several authors [9,23,57] our first hypothesis was confirmed: scores for expected answers were higher in the comprehension than in the production task.

We found an age effect in the production task with a significant increase in the mean scores after the two estimated breakpoints of 8 and 10 years old. Unlike the comprehension task, in the production task we found a significant difference between scores according to the difficulty levels of sentences. This task was well succeeded for D1 sentences with 84.5% of expected answers unlike for D2 sentences for which we found 7.5% expected answers. Fig 4 illustrates this difference in performances in production between D1 and D2, as well as the increase in the mean scores found at 8 and 10 years.

For D1, we found higher scores for expected answers in comparison with scores for expected answers with change of perspective and with scores for unexpected answers. Conversely, in D2 we found a majority of unexpected answers, in comparison with expected answers with and without change of perspective. As expected, regarding a possible persistent structural priming effect from comprehension to production [58], most answers in the production task were either expected or unexpected answers (CF. Table 5). However, the priming effect could be moderated by the fact that, even if perspectives for each picture were similar, gender of PersPs were different between the two tasks.

In the production task, the unexpected answers for D1 sentences involved more the DO clitics (relating first to the use of gender) than the S. These results are in line with the idea that, in a developmental perspective, S clitics emerge very early amongst French-speaking children, while object clitics are delayed by several months [8,9,29,52,59]. Our results are also in accordance with the pronominal clitics complexity scale of Prevost (2018) [60] where subject clitics are at the bottom (i.e., the easiest) and accusative clitics are the most complex elements. In this regard, contrary to S clitics, object clitics occur before the verb in French, which disrupt the canonical SVO word order occurring in a pre-posed position (See introduction). In addition, DO include 3p which was the most frequent perspective used in our protocol (Table 3). 3p DO are marked for both number and gender and their reference, in contrast to 1p and 2p, established via discourse [60]. In D1 sentences, conversely for S, we found lower scores of expected answers in 3p than in 1p DO.

Comparing the production of IO and DO in French-speaking children from 3 to 5 years of age, Bello & Pirvulescu (2022) [33] found that IO clitics appear later than DO clitics. However, they used sentences with either DO or IO. In our protocol, D2 sentences included both DO and IO.

Our results indicated that unexpected answers involved, in decreasing order, scores for DO, then for IO, and finally for S. Concerning unexpected answers on DO, we found that they were related first on the omission of DO and then on the non-pronominalization of DO. For IO, unexpected answers related mainly on the omission of IO. In the production task, an explanation regarding significantly lower scores for D2 compared to D1, could be the number and the similarities of the PersP’s characteristics. Several authors argue that to avoid ambiguity, listeners tend to use less PersP than Noun-Phrases in contexts where a target referent is elicited in the presence of a competitor [61–63]. The avoidance of PersP would help listeners discriminate similar referents. The authors formulated the competition in terms of linguistic (gender) or non-linguistic (animate or inanimate) similarity.

Concerning the relationship between the comprehension task and the production task, regarding Fig 4, performances in D1 for the two tasks seem to be related, even for the youngster children, which is in line with those of the study of Veneziano (2022) [13] that involved children of 2 years old. In D2, conversely, it appears the results between the two tasks are dissociated. In the production task, even if their performances tend to increase with age, the production of D2 sentences remains difficult even among the children after 10 years.

These difficulties in D2 sentences could be explained from a grammatical and a pragmatic point of view. Syntactically, D2 sentences are more complex than D1 sentences on the fact that they involve two object complements (DO and IO). They combined the disruption of the canonical order SVO with the use of two different configurations, S DO IO (in the 1p and 2p perspectives) and S IO DO (in the 3p perspective).

Furthermore, this complexity, classically measured in terms of mean length of utterance in words, is well known to be related to a variety of syntactic milestones as well as to the role of cognitive systems [64,65]. PersP interpretation is underpinned by several cognitive mechanisms, in particular inhibition skills and theory of mind abilities of the participants [51,66,67].

From a pragmatic point of view, children had to embody the point of view of several characters (the speaker, the addressee, and the observer). We tested their ability to shift and integrate perspectives between different speech roles in various contexts involving S, DO and IO. Furthermore, taking the other’s perspective relies on the ability to move from an embodied to a disembodied perspective, to coordinate multiple perspectives in one coherent spatial framework [68].

Concerning expected answers, we identified a significant increase in the mean scores after an estimated breakpoint of 8 years for the comprehension task and, after the two estimated breakpoints of 8 and 10 years for the production task. Our findings related to perspective-taking ability from a language point of view, are consistent with those of (1) Piaget (1967) [47] and (2) other studies exploring this cognitive ability from a visual and/ or a motor point of view [69–70].

In D2 sentences, due to a greater computational complexity, we could argue that children would focus primarily on the partners of the interaction involved in the speech context (designated by S and IO), rather than on the object of the interaction (DO).

PersP involves language aspects as well as the ability for children to put him/herself in other people’ shoes. The latter is an important component of social cognition. This type of protocol could constitute a suitable tool for assessment as well as training of PersP’s use, in a school context. Furthermore, in France there is no existing specific tools for the assessment of PersP. Therefore, it would be interesting to use this tool amongst children with neurodevelopmental disorders, especially with language disorder.

### Study limitations

The results of the current study should be interpreted according to the study limitations. First, the number of PersP was somewhat restricted and particularly in terms of 2p. Furthermore, we only had 3p in DO for D2 sentences.

According to the two existing configurations in D2 sentences, it would be interesting to use the same number of sentences in each configuration, S DO IO and S IO DO.

It would also be important, even if difficult to control, to consider the input that children receive from their caregivers. In typical development, the quantity and quality of input predicts language ability [71]. According to Lieven (2010) [72] and Clark [73], the more frequently children hear a particular word or construction, the earlier they acquire it.

## Conclusions

We explored the acquisition of PersP in children from 6 to 13 years old, using a protocol consisting of various ecological situations of the children’s daily life, in a comic strip format. Following a developmental perspective, we used the comprehension task before the production task and sentences with two levels of syntactic difficulty.

After the age of six, children’s performances in the acquisition of PersP, in comprehension and in production, increase with age. We identified a significant increase in the mean scores of expected answers after an estimated breakpoint of 8 years for the comprehension task and after the two estimated breakpoints of 8 and 10 years for the production task. In this task, children had difficulties with sentences including two objects complement. These difficulties could be explained by the computational complexity of the production task in terms of morphosyntactic and pragmatic constraints. Furthermore, difficulties were focused on the use of object pronouns, and mainly DO. We could suggest that, in D2 sentences, children focus primarily on the partners of the interaction involved in the speech context (designated by S and IO), rather than on the object of the interaction (DO). Further research are needed to assess this hypothesis.

Therefore, it would be interesting to use this tool amongst children with neurodevelopmental disorders, specifically with language disorder. We could assess their performances and compare the results with those obtained by our group of typically developing children in our study. This type of protocol could be used amongst children with language disorders in sessions of speech therapy.

## Supporting information

S1 File The tasks in French language. (DOCX)

S2 File The tasks in English language. (DOCX)
