# Supplementary material for: The acquisition of personal pronouns’ comprehension and production in French-speaking children: Toward the ability to embody characters’ perspectives in various pictured speech interactions
Source: PLoS One. 2025 Sep 22;20(9):e0324218. doi: 10.1371/journal.pone.0324218 (PMC12453247; doi:10.1371/journal.pone.0324218)
Supplement: S1 File — (DOCX) [file pone.0324218.s001.docx]

**Tâches de compréhension et de production des pronoms personnels**

# Ce protocole expérimental consiste en la présentation de plusieurs situations sociales, par le biais d’une image.

# Chaque fois qu’une image est montrée, l’évaluateur donne les instructions, tout en pointant les différents personnages du doigt au moment où il les mentionne.

#

# Tâche de compréhension



# Commencer avec un exemple est important, car cela va aider le participant à comprendre ce qui est attendu. Ne pas hésiter à répéter et à reformuler.

## Exemple


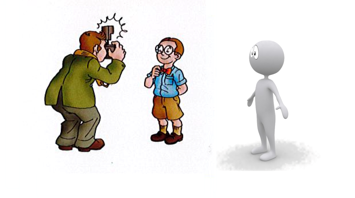


Sur cette image, on voit un garçon (*montrer le garçon*), un photographe (*montrer le photographe*) et une personne qui les regarde (*montrer la personne qui les regarde*). Chaque personne peut parler. Je vais lire des phrases. Peux-tu me montrer qui dit ?

|  | ***Phrase à dire au participant*** | Le garçon | Le photographe | La personne qui regarde |
| --- | --- | --- | --- | --- |
| **Phrase 1** | **« Je le prends en photo »** |  | **1** |  |
| **Phrase 2** | **« Il te prend en photo »** |  |  | **1** |
| **Phrase 3** | **« Il me prend en photo »** | **1** |  |  |

## La balançoire

##
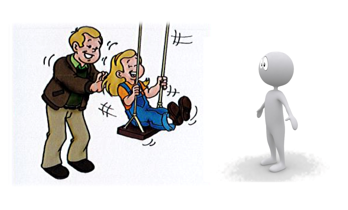


Sur cette image, on voit une fille (*montrer la fille*), son père (*montrer son père*), et quelqu’un qui les regarde (*montrer la personne qui les regarde*). Ils sont dans un parc. La petite fille est sur la balançoire. Chaque personne peut parler. Je vais lire des phrases. Peux-tu me montrer qui dit ?

|  | ***Phrase à dire au participant*** | La fille | Le père | La personne qui regarde |
| --- | --- | --- | --- | --- |
| **Phrase 1** | **« Il la pousse »** |  |  | **1** |
| **Phrase 2** | **« Il me pousse »** | **1** |  |  |
| **Phrase 3** | **« Je la pousse »** |  | **1** |  |

## La cérémonie de remise des médailles


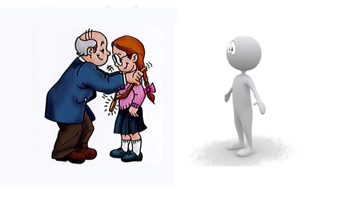


Sur cette image, on voit une fille (*montrer la fille*), le directeur de l’école (*montrer le directeur*), et quelqu’un qui les regarde (*montrer la personne qui les regarde*). Le directeur remet la médaille à la fille. Chaque personne peut parler. Je vais lire des phrases. Peux-tu me montrer qui dit ?

|  | ***Phrases à dire au participant*** | La fille | Le directeur | La personne qui regarde |
| --- | --- | --- | --- | --- |
| **Phrase 1** | **« Il me la remet »** | **1** |  |  |
| **Phrase 2** | **« Je te la remets »** |  | **1** |  |
| **Phrase 3** | **« Il la lui remet »** |  |  | **1** |

## Mauvais comportement


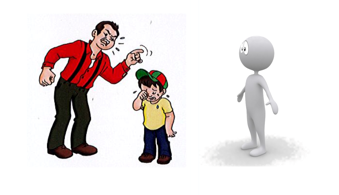


Sur cette image, on voit un garçon (*montrer le garçon*), son père (*montrer le père*), et quelqu’un qui les regarde (*montrer la personne qui les regarde*). Le garçon s’est mal comporté. Chaque personne peut parler. Je vais lire des phrases. Peux-tu me montrer qui dit ?

|  | ***Phrases à dire au participant*** | Le garçon | Le père | La personne qui regarde |
| --- | --- | --- | --- | --- |
| **Sentence 1** | **« Je le gronde »** |  | **1** |  |
| **Sentence 2** | **« Il me gronde »** | **1** |  |  |
| **Sentence 3** | **« Il le gronde »** |  |  | **1** |

## En balade à l’extérieur


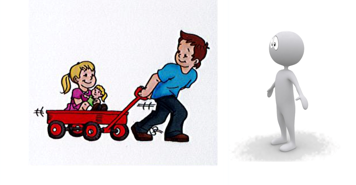


Sur cette image, on voit un grand frère (*montrer le grand frère*), sa petite sœur (*montrer la petite sœur*) et quelqu’un qui les regarde (*montrer la personne qui les regarde*). Ils sont en train de se balader dehors. Chaque personne peut parler. Je vais lire des phrases. Peux-tu me montrer qui dit ?

|  | ***Phrases à dire au participant*** | Le grand frère | La petite sœur | La personne qui regarde |
| --- | --- | --- | --- | --- |
| **Phrase 1** | **« Il me tire »** |  | **1** |  |
| **Phrase 2** | **« Il la tire »** |  |  | **1** |
| **Phrase 3** | **« Je la tire »** | **1** |  |  |

## L’heure du coucher


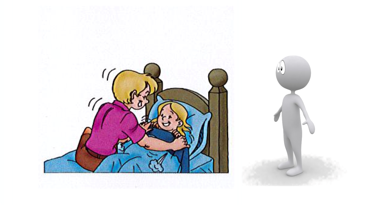


Sur cette image, on voit une fille (*montrer la fille*), sa mère (*montrer la mère*), et quelqu’un qui regarde (*montrer la personne qui regarde*). C’est l’heure du coucher. La mère borde la fille en lui disant « Bonne nuit ». Chaque personne peut parler. Je vais lire des phrases. Peux-tu me montrer qui dit ?

|  | ***Phrases à dire au participant*** | La mère | La fille | La personne qui regarde |
| --- | --- | --- | --- | --- |
| **Phrase 1** | **« Elle la borde »** |  |  | **1** |
| **Phrase 2** | **« Je la borde »** | **1** |  |  |
| **Phrase 3** | **« Elle me borde »** |  | **1** |  |

## Chez le coiffeur


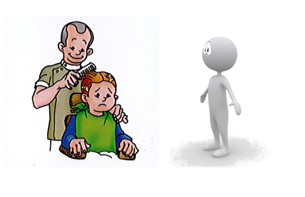


Sur cette image, on voit un garçon (*montrer le garçon*), un coiffeur (*montrer le coiffeur*), et quelqu’un qui les regarde (*montrer la personne qui les regarde*). Le coiffeur coiffe les cheveux du garçon. Chaque personne peut parler. Je vais lire des phrases. Peux-tu me montrer qui dit ?

|  | ***Phrases à dire au participant*** | Le garçon | Le coiffeur | La personne qui regarde |
| --- | --- | --- | --- | --- |
| **Phrase 1** | **« Il les lui coiffe»** |  |  | **1** |
| **Phrase 2** | **« Il me les coiffe »** | **1** |  |  |
| **Phrase 3** | **« Je les lui coiffe »** |  | **1** |  |

## L’heure du bain


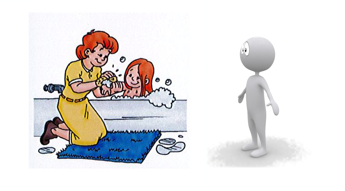


Sur cette image, on voit une petite fille (*montrer la petite fille*), sa mère (*montrer la mère*), et quelqu’un qui les regarde (*montrer la personne qui les regarde*). La petite fille est dans le bain. Ses mains sont sales. Chaque personne peut parler. Je vais lire des phrases. Peux-tu me montrer qui dit ?

|  | ***Phrases à dire au participant*** | La mère | La fille | La personne qui regarde |
| --- | --- | --- | --- | --- |
| **Phrase 1** | **« Tu me les laves »** |  | **1** |  |
| **Phrase 2** | **« Je te les lave»** | **1** |  |  |
| **Phrase 3** | **« Elle les lui lave»** |  |  | **1** |

## Le portrait


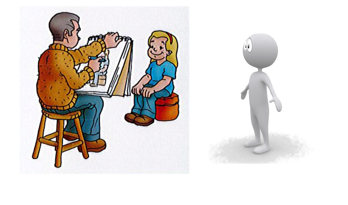


Sur cette image, on voit une fille (*montrer la fille*), un peintre (*montrer le peintre*), et quelqu’un qui les regarde (*montrer la personne qui les regarde*). Le peintre dessine un portrait de la fille. Chaque personne peut parler. Je vais lire des phrases. Peux-tu me montrer qui dit ?

|  | ***Phrases à dire au participant*** | Le peintre | La petite fille | La personne qui regarde |
| --- | --- | --- | --- | --- |
| **Phrase 1** | **« Je te le dessine»** | **1** |  |  |
| **Phrase 2** | **« Il le lui dessine»** |  |  | **1** |
| **Phrase 3** | **« Il me le dessine»** |  | **1** |  |

## Carnaval


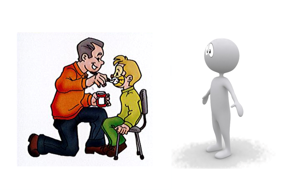


Sur cette image, on voit un garçon (*montrer le garçon*), un animateur (*montrer l’animateur*), et quelqu’un qui les regarde (*montrer la personne qui les regarde*). C’est Carnaval. L’animateur maquille le garçon. Chaque personne peut parler. Je vais lire des phrases. Peux-tu me montrer qui dit ?

|  | ***Phrases à dire au participant*** | L’animateur | Le garçon | La personne qui regarde |
| --- | --- | --- | --- | --- |
| **Phrase 1** | **« Il me maquille »** |  | **1** |  |
| **Phrase 2** | **« Il le maquille »** |  |  | **1** |
| **Phrase 3** | **« Je le maquille »** | **1** |  |  |

## Au restaurant


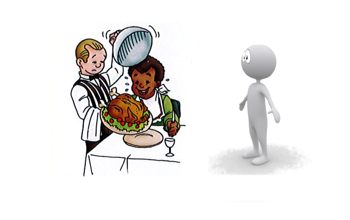


Sur cette image, on voit un client (*montrer le client*), un serveur (*montrer le serveur*), et quelqu’un qui les regarde (*montrer la personne qui les regarde*). Le client a commandé du poulet. Chaque personne peut parler. Je vais lire des phrases. Peux-tu me montrer qui dit ?

|  | ***Phrases à dire au participant*** | Le serveur | Le client | La personne qui regarde |
| --- | --- | --- | --- | --- |
| **Phrase 1** | **« Il le lui sert »** |  |  | **1** |
| **Phrase 2** | **« Je te le sers »** | **1** |  |  |
| **Phrase 3** | **« Il me le sert »** |  | **1** |  |

## Tâche de production

Dans cet exercice, le démarrage par l’exemple est plus important encore, car les participants pourraient spontanément formuler des réponses hors de propos. Ils doivent comprendre ce qui est attendu d’eux exactement. Ne pas hésiter à répéter et à reformuler.

- **Exemple**


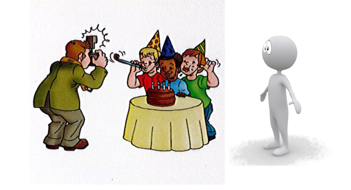


Sur cette image, on voit des enfants (*montrer les enfants*), un photographe (*montrer le photographe*), et quelqu’un qui les regarde (*montrer la personne qui les regarde*). Que dit :

|  | « Je les prends en photo» | « Il les prend en photo » | « Il nous prend en photo » |
| --- | --- | --- | --- |
| **Le photographe** | **1** |  |  |
| **L’enfant** |  |  | **1** |
| **La personne qui regarde** |  | **1** |  |

## La balançoire


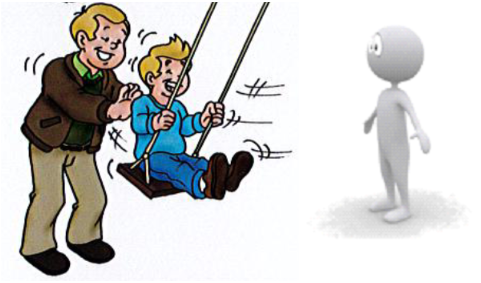


Sur cette image, on voit un petit garçon (*montrer le garçon*), son père (*montrer le père*) et quelqu’un qui les regarde (*montrer la personne qui les regarde*). Ils sont dans un parc. Le petit garçon est sur la balançoire. Que dit :

|  | « Il le pousse » | « Il me pousse » | « Je le pousse » |
| --- | --- | --- | --- |
| **Le petit garçon** |  | **1** |  |
| **Le père** |  |  | **1** |
| **La personne qui regarde** | **1** |  |  |

## La cérémonie de remise des médailles


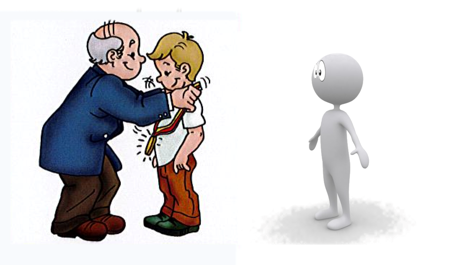


Sur cette image, on voit un garçon (*montrer le garçon*), le directeur de l’école (*montrer le directeur*) et quelqu’un qui les regarde (*montrer la personne qui les regarde*). Le directeur remet la médaille au garçon. Que dit :

|  | « Il me la remet » | « Je te la remets » | « Il la lui remet » |
| --- | --- | --- | --- |
| **Le directeur** |  | **1** |  |
| **La personne qui regarde** |  |  | **1** |
| **Le garçon** | **1** |  |  |

## Mauvais comportement


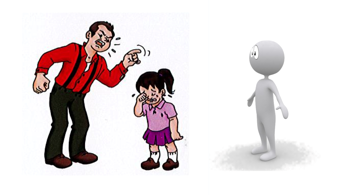


Sur cette image, on voit une fille (*montrer la fille*), son père (*montrer le père*) et quelqu’un qui les regarde (*montrer la personne qui les regarde*). La fille s’est mal comportée. Que dit :

|  | « Je la gronde » | « Il me gronde » | « Il la gronde » |
| --- | --- | --- | --- |
| **La personne qui regarde** |  |  | **1** |
| **Le fille** |  | **1** |  |
| **Le père** | **1** |  |  |

## En balade à l’extérieur


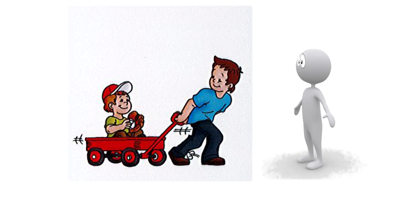


Sur cette image, on voit un grand frère (*montrer le grand frère*), son petit frère (*montrer le petit frère*), et quelqu’un qui les regarde (*montrer la personne qui les regarde*). Ils se baladent à l’extérieur. Le petit frère est assis dans une charrette. Que dit :

|  | « Il le tire » | « Je le tire » | « Il me tire » |
| --- | --- | --- | --- |
| **La personne qui regarde** | **1** |  |  |
| **Le grand frère** |  | **1** |  |
| **Le petit frère** |  |  | **1** |

## L’heure du coucher


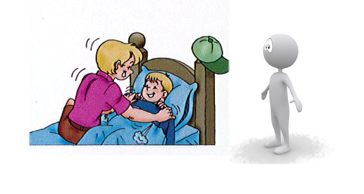


Sur cette image, on voit un garçon (*montrer le garçon*), sa mère (*montrer la mère*), et quelqu’un qui les regarde (*montrer la personne qui les regarde*). C’est l’heure du coucher. La mère borde le garçon avant de lui dire bonne nuit. Que dit :

|  | « Je le borde » | « Elle me borde » | « Elle le borde » |
| --- | --- | --- | --- |
| **La mère** | **1** |  |  |
| **Le garçon** |  | **1** |  |
| **La personne qui regarde** |  |  | **1** |

## Chez le coiffeur


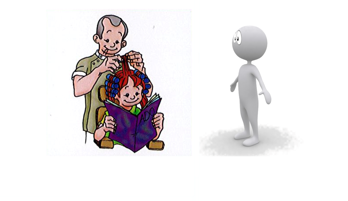


Sur cette image, on voit une fille (*montrer la fille*), un coiffeur (*montrer le coiffeur*), et quelqu’un qui les regarde (*montrer la personne qui les regarde*). Le coiffeur coiffe les cheveux de la fille. Que dit :

|  | « Je te les coiffe » | « Il les lui coiffe» | « Il me les coiffe» |
| --- | --- | --- | --- |
| **La fille** |  |  | **1** |
| **La personne qui regarde** |  | **1** |  |
| **Le coiffeur** | **1** |  |  |

## L’heure du bain


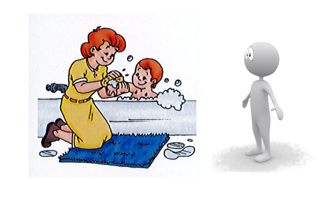


Sur cette image, on voit un petit garçon (*montrer le petit garçon*), sa mère (*montrer la mère*), et quelqu’un qui les regarde (*montrer la personne qui les regarde*). Le petit garçon est dans le bain. Ses mains sont sales. Que dit:

|  | « Je te les lave» | « Elle me les lave » | « Elle les lui lave » |
| --- | --- | --- | --- |
| **La mère** | **1** |  |  |
| **La personne qui regarde** |  |  | **1** |
| **Le garçon** |  | **1** |  |

## Le portrait


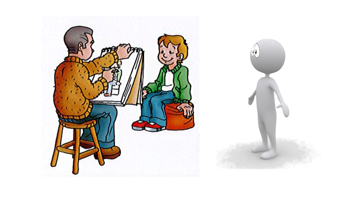


Sur cette image, on voit un garçon (*montrer le garçon*), un peintre (*montrer le peintre*), et quelqu’un qui les regarde (*montrer la personne qui les regarde*). Le peintre dessine un portrait pour le garçon. Que dit:

|  | « Je le lui dessine» | « Il me le dessine | « Il le lui dessine » |
| --- | --- | --- | --- |
| **Le garçon** |  | **1** |  |
| **Le peintre** | **1** |  |  |
| **La personne qui regarde** |  |  | **1** |

## Carnaval


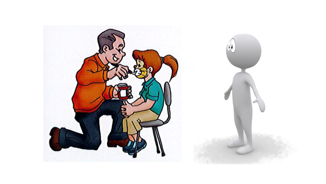


Sur cette image, on voit une fille (*montrer la fille*), un animateur (*montrer l’animateur*), et quelqu’un qui les regarde (*montrer la personne qui les regarde*). C’est Carnaval. L’animateur maquille la fille. Que dit :

|  | « Je la maquille » | « Il me maquille » | « Il la maquille » |
| --- | --- | --- | --- |
| **L’animateur** | **1** |  |  |
| **La fille** |  | **1** |  |
| **La personne qui regarde** |  |  | **1** |

## Au restaurant


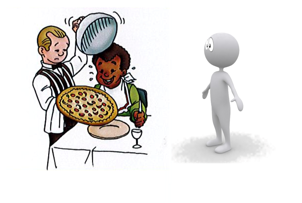


Sur cette image, tu peux voir un client (*montrer le client*), un serveur (*montrer le serveur*) et quelqu’un qui les regarde (*montrer la personne qui les regarde*). Le client a commandé une pizza. Que dit :

|  | « Je te le sers » | « Il la lui sert » | « Il me la sert » |
| --- | --- | --- | --- |
| **Le serveur** | **1** |  |  |
| **Le client** |  |  | **1** |
| **La personne qui regarde** |  | **1** |  |
