# Supplementary material for: The acquisition of personal pronouns’ comprehension and production in French-speaking children: Toward the ability to embody characters’ perspectives in various pictured speech interactions
Source: PLoS One. 2025 Sep 22;20(9):e0324218. doi: 10.1371/journal.pone.0324218 (PMC12453247; doi:10.1371/journal.pone.0324218)
Supplement: S2 File — (DOCX) [file pone.0324218.s002.docx]

**Comprehension and production tasks of PP**

# The experimental protocol consisting of various speech contexts in a comic strip format

Each time a picture is shown, the evaluator gives instructions, while pointing to the different characters as he mentions them.

# Comprehension task



Starting with an example is important, as it will help the participant to understand what is expected. Do not hesitate to repeat and rephrase.

## Example


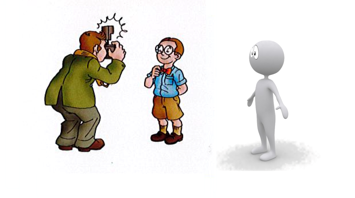


In this picture you can see a boy (*show the boy*), a photographer (*show the photographer*) and someone watching (*show the person watching*). Each person can speak. Show me who is saying ?

|  | ***Sentences to say to the participant*** | The boy | The photographer | The person watching |
| --- | --- | --- | --- | --- |
| **Sentence 1** | **‘I’m taking a picture of him’** |  | **1** |  |
| **Sentence 2** | **‘He’s taking a picture of him’** |  |  | **1** |
| **Sentence 3** | **‘He is taking a picture of me’** | **1** |  |  |

## The swing

##
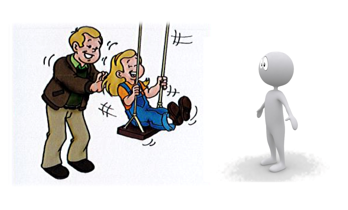


In this picture you can see a little girl (*show the little girl*), her father (*show the father*) and someone watching (*show the person watching*). They are in a park. The little girl is on the swing. Each person can speak. Show me who is saying ?

|  | ***Sentences to say to the participant*** | The little girl | The father | The person watching |
| --- | --- | --- | --- | --- |
| **Sentence 1** | **‘He is pushing her’** |  |  | **1** |
| **Sentence 2** | **‘He is pushing me’** | **1** |  |  |
| **Sentence 3** | **‘I’m pushing her’** |  | **1** |  |

## The medal ceremony


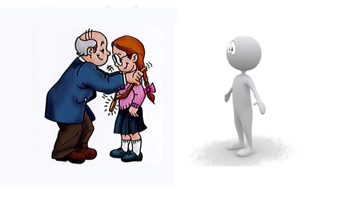


In this picture you can see a girl (*show the girl*), the headmaster of her school (*show the headmaster*) and someone watching (*show the person watching*). The headmaster is awarding a medal to the girl. Each person can speak. Show me who is saying ?

|  | ***Sentences to say to the participant*** | The girl | The Headmaster | The person watching |
| --- | --- | --- | --- | --- |
| **Sentence 1** | **‘He is awarding it to me’** | **1** |  |  |
| **Sentence 2** | **‘I’m awarding it to you’** |  | **1** |  |
| **Sentence 3** | **‘He is awarding it to her’** |  |  | **1** |

## The misbehaving


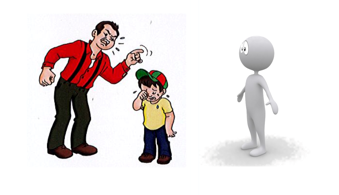


In this picture, you can see a boy (*show the boy*), his father (*show the father*) and someone watching (*show the person watching*). The boy has misbehaved. Each person can speak. Show me who is saying?

|  | ***Sentences to say to the participant*** | The boy | The father | The person watching |
| --- | --- | --- | --- | --- |
| **Sentence 1** | **‘I’m telling him off’** |  | **1** |  |
| **Sentence 2** | **‘He is telling me off’** | **1** |  |  |
| **Sentence 3** | **‘He is telling him off’** |  |  | **1** |

## Out on a walk


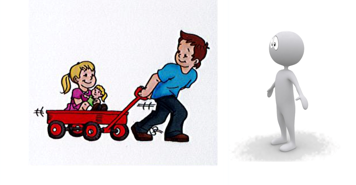


In this picture you can see a big brother (*show the big brother*), his little sister (*show the little sister)* and someone watching (*show the person watching*). They are out on a walk. The little sister is sitting in a kind of car on wheels. Each person can speak. Show me who is saying?

|  | ***Sentences to say to the participant*** | The big brother | The little sister | The person watching |
| --- | --- | --- | --- | --- |
| **Sentence 1** | **‘He is pulling me’** |  | **1** |  |
| **Sentence 2** | **‘He is pulling her’** |  |  | **1** |
| **Sentence 3** | **‘I’m pulling her’** | **1** |  |  |

## Bedtime


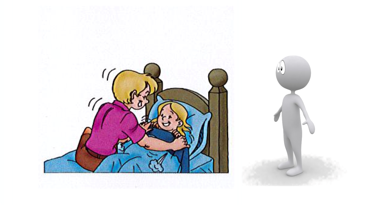


In this picture you can see a girl (*show the girl*), her mum (*show the mum*) and someone watching (*show the person watching*). It's bedtime. The mummy is tucking the little girl in before saying "good night". Each person can speak. Show me who is saying?

|  | ***Sentences to say to the participant*** | The mother | The daughter | The person watching |
| --- | --- | --- | --- | --- |
| **Sentence 1** | **‘She is tucking her in’** |  |  | **1** |
| **Sentence 2** | **‘I’m tucking her in’** | **1** |  |  |
| **Sentence 3** | **‘She is tucking me in’** |  | **1** |  |

## At the hairdresser


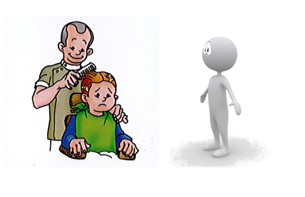


In this picture you can see a boy (*show the boy*), a hairdresser (*show the hairdresser*) and someone watching (*show the person watching*). The hairdresser is styling the boy's hair. Each person can speak. Show me who is saying?

|  | ***Sentences to say to the participant*** | The boy | The hairdresser | The person watching |
| --- | --- | --- | --- | --- |
| **Sentence 1** | **‘He is styling his hair’** |  |  | **1** |
| **Sentence 2** | **‘He is styling my hair’** | **1** |  |  |
| **Sentence 3** | **‘I’m styling your hair’** |  | **1** |  |

## Bath time


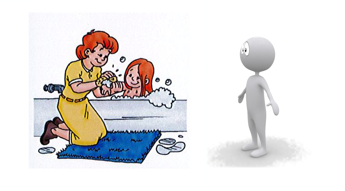


In this picture you can see a little girl (*show the little girl*), her mum (*show the mum*) and someone watching (*show* *the person watching*). The little girl is in the bath. Her hands are dirty. Each person can speak. Show me who is saying?

|  | ***Sentences to say to the participant*** | The mother | The daughter | The person watching |
| --- | --- | --- | --- | --- |
| **Sentence 1** | **‘You are washing them for me’** |  | **1** |  |
| **Sentence 2** | **‘I’m washing them for you’** | **1** |  |  |
| **Sentence 3** | **‘She is washing them for her’** |  |  | **1** |

## The portrait


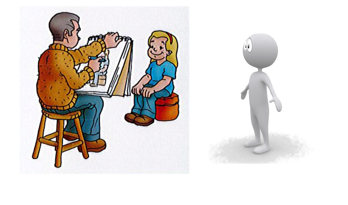


In this picture you can see there is a girl (*show the girl*), there is a painter (*show the painter*) and there is someone looking (*show the person looking*). The painter is drawing a portrait of the girl. Each person can speak. Show me who is saying?

|  | ***Sentences to say to the participant*** | The painter | The little girl | The person watching |
| --- | --- | --- | --- | --- |
| **Sentence 1** | **‘I’m drawing it for her’** | **1** |  |  |
| **Sentence 2** | **‘He is drawing it for her’** |  |  | **1** |
| **Sentence 3** | **‘He is drawing it for me’** |  | **1** |  |

## At the carnival


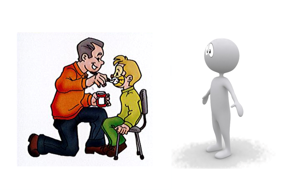


In this picture you can see a boy (*show the boy*), there's an activity leader (*show the entertainer*) and there's someone watching (*show the person watching*). It's carnival time. The activity leader is putting make-up on the boy. Each person can speak. Show me who is saying?

|  | ***Sentences to say to the participant*** | The activity leader | The boy | The person watching |
| --- | --- | --- | --- | --- |
| **Sentence 1** | **‘He is doing my makeup’** |  | **1** |  |
| **Sentence 2** | **‘He is doing his makeup’** |  |  | **1** |
| **Sentence 3** | **‘I’m doing his makeup’** | **1** |  |  |

## At the restaurant


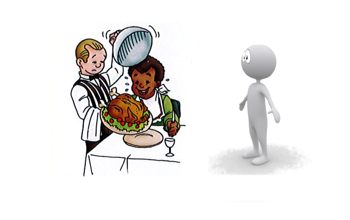


In this picture you can see a customer (*show the customer*), a waiter (*show the waiter*) and someone watching (*show the person watching*). The customer ordered a chicken. Each person can speak. Show me who is saying?

|  | ***Sentences to say to the participant*** | The waiter | The customer | The person watching |
| --- | --- | --- | --- | --- |
| **Sentence 1** | **‘He is serving it to him’** |  |  | **1** |
| **Sentence 2** | **‘I’m serving it to you’** | **1** |  |  |
| **Sentence 3** | **‘He is serving it to me’** |  | **1** |  |

## Production task

In this case, starting by an example is even more important, as participants may spontaneously formulate out-of-context answers. They need to understand what is expected. Do not hesitate to repeat and rephrase.

**Example**


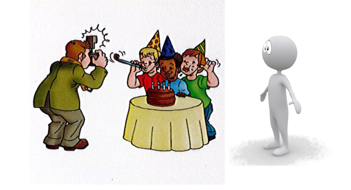


In this picture you can see children (*show the children*), a photographer (*show the photographer*) and someone watching (*show the person watching*). What do they say?

|  | ‘I’m taking a picture of them’ | ‘He is taking a picture of them’ | ‘He is taking a picture of us’ |
| --- | --- | --- | --- |
| **The photographer** | **1** |  |  |
| **The children** |  |  | **1** |
| **The person watching** |  | **1** |  |

## The swing


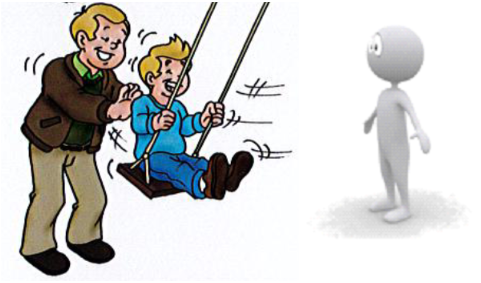


In this picture you can see a little boy (*show the boy*), his father (*show the father*) and someone watching (*show the person watching*). They are in a park. The little boy is on the swing. What do they say ?

|  | ‘He is pushing him’ | ‘He is pushing me’ | ‘I’m pushing him’ |
| --- | --- | --- | --- |
| **The little boy** |  | **1** |  |
| **The father** |  |  | **1** |
| **The person watching** | **1** |  |  |

## The medal ceremony


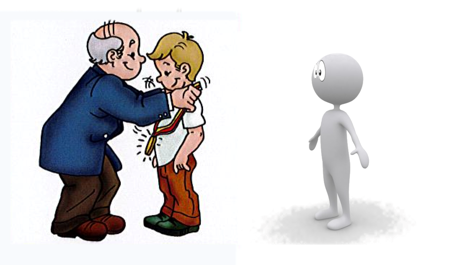


In this picture you can see a boy (*show the boy*), the headmaster of his school (*show the headmaster*) and someone watching (*show the person watching*). The headmaster is awarding the boy a medal. What do they say?

|  | ‘He is awarding it to me’ | ‘I’m awarding it to you’ | ‘He is awarding it to him’ |
| --- | --- | --- | --- |
| **The Headmaster** |  | **1** |  |
| **The person watching** |  |  | **1** |
| **The boy** | **1** |  |  |

## The misbehaving


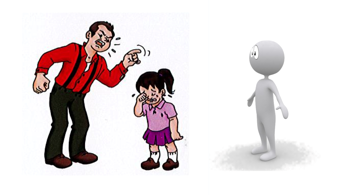


In this picture you can see a girl (*show the girl*), her dad (*show the dad*) and someone watching (*show the person watching*). The girl has misbehaved. What do they say?

|  | ‘I’m telling her off’ | ‘He is telling me off’ | ‘He is telling her off’ |
| --- | --- | --- | --- |
| **The person watching** |  |  | **1** |
| **The girl** |  | **1** |  |
| **The father** | **1** |  |  |

## Out on a walk


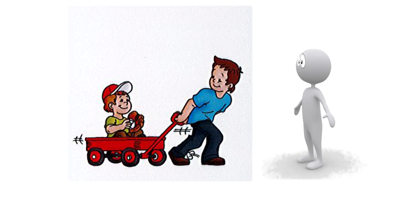


In this picture you can see a big brother (*show the big brother*), his little brother (*show the little brother*) and someone watching (*show the person watching*). They are out on a walk. The little brother is sitting in a kind of car with wheels. What do they say?

|  | ‘He is pulling him’ | ‘I’m pulling him’ | ‘He is pulling me’ |
| --- | --- | --- | --- |
| **The person watching** | **1** |  |  |
| **The big brother** |  | **1** |  |
| **The little brother** |  |  | **1** |

## Bedtime


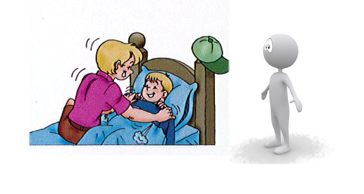


In this picture you can see a boy (*show the boy*), his mum (*show the mum*) and someone watching (*show the person watching*). It's bedtime. The mum is tucking the little boy in before saying "good night". What do they say?

|  | ‘I’m tucking him in’ | ‘She is tucking me in’ | ‘She is tucking him in’ |
| --- | --- | --- | --- |
| **The mother** | **1** |  |  |
| **The boy** |  | **1** |  |
| **The person watching** |  |  | **1** |

## At the hairdresser’s


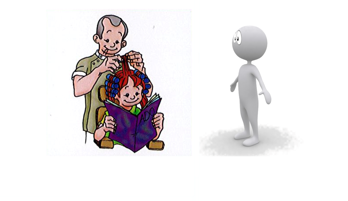


In this picture you can see a girl (*show the girl*), a hairdresser (*show the hairdresser*) and someone watching (*show the person watching*). The hairdresser is styling the girl's hair. What do they say?

|  | ‘I’m styling it for you’ | ‘He is styling it for her ’ | ‘He is styling it for me’ |
| --- | --- | --- | --- |
| **The girl** |  |  | **1** |
| **The person watching** |  | **1** |  |
| **The hairdresser** | **1** |  |  |

## Bath time


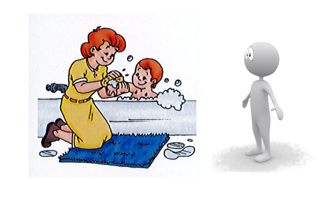


In this picture you can see a little boy (*show the little boy*), his mum (*show the mum*) and someone watching (*show the person watching*). The little boy is in the bath. His hands are dirty. What do they say?

|  | ‘I’m washing them for you’ | ‘She is washing them for me’ | ‘She is washing them for him’ |
| --- | --- | --- | --- |
| **The mother** | **1** |  |  |
| **The person watching** |  |  | **1** |
| **The boy** |  | **1** |  |

## The portrait


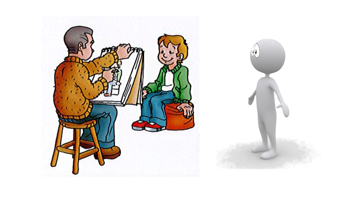


In this picture you can see there is a boy (*show the boy*), there is a painter (*show the painter*) and there is someone looking (*show the person looking*). The painter is drawing a portrait of the boy. What do they say?

|  | ‘I’m drawing it for him’ | ‘He is drawing it for me’ | ‘He is drawing it for him’ |
| --- | --- | --- | --- |
| **The boy** |  | **1** |  |
| **The painter** | **1** |  |  |
| **The person watching** |  |  | **1** |

## At the carnival


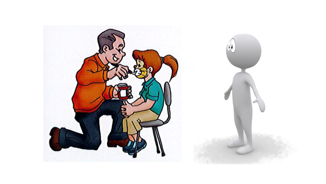


In this picture you can see a girl (*show the girl*), there's an activity leader (*show the activity leader*) and there's someone watching (*showing the person watching*). It's carnival time. The activity leader is putting make-up on the girl. What do they say?

|  | ‘I’m doing her makeup’ | ‘He is doing my makeup’ | ‘He is doing her makeup’ |
| --- | --- | --- | --- |
| **The activity leader** | **1** |  |  |
| **The girl** |  | **1** |  |
| **The person watching** |  |  | **1** |

## At the restaurant


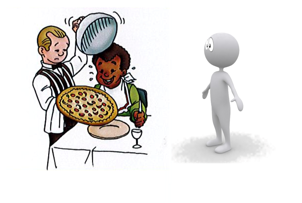


In this picture you can see a customer (*show the customer*), a waiter (*show the waiter*) and someone watching (*show the person watching*). The man ordered a pizza. What do they say?

|  | ‘I’m serving it to you’ | ‘He is serving it to him’ | ‘He is serving it to me’ |
| --- | --- | --- | --- |
| **The waiter** | **1** |  |  |
| **The customer** |  |  | **1** |
| **The person watching** |  | **1** |  |
